# Supplementary material for: Connectome-harmonic decomposition of human brain activity reveals dynamical repertoire re-organization under LSD
Source: Sci Rep. 2017 Dec 15;7:17661. doi: 10.1038/s41598-017-17546-0 (PMC5732294; doi:10.1038/s41598-017-17546-0)
Supplement: Supplementary file 1 — Supplementary figures [file 41598_2017_17546_MOESM1_ESM.pdf]

Supplementary figures for Connectome-harmonic decomposition of human brain activity reveals dynamical repertoire re-organization under LSD

Selen Atasoy[1,*], Leor Roseman[2], Mendel Kaelen[2], Morten L. Kringelbach[3,4], Gustavo Deco[1,5,6,7], and Robin L. Carhart-Harris[2]

[1]Center of Brain and Cognition, Universitat Pompeu Fabra, Barcelona, Spain
[2]Psychedelic Research Group, Centre for Psychiatry, Division of Brain Sciences, Imperial College London
[3]Department of Psychiatry, University of Oxford
[4]Center for Music in the Brain, Aarhus University, Denmark
[5]ICREA, Institució Catalana de Recerca i Estudis Avancats (ICREA), Spain
[6]Department of Neuropsychology, Max Planck Institute for Human Cognitive and Brain Sciences, Leipzig, Germany
[7]School of Psychological Sciences, Monash University, Melbourne, Australia
[*]selenatasoy@gmail.com

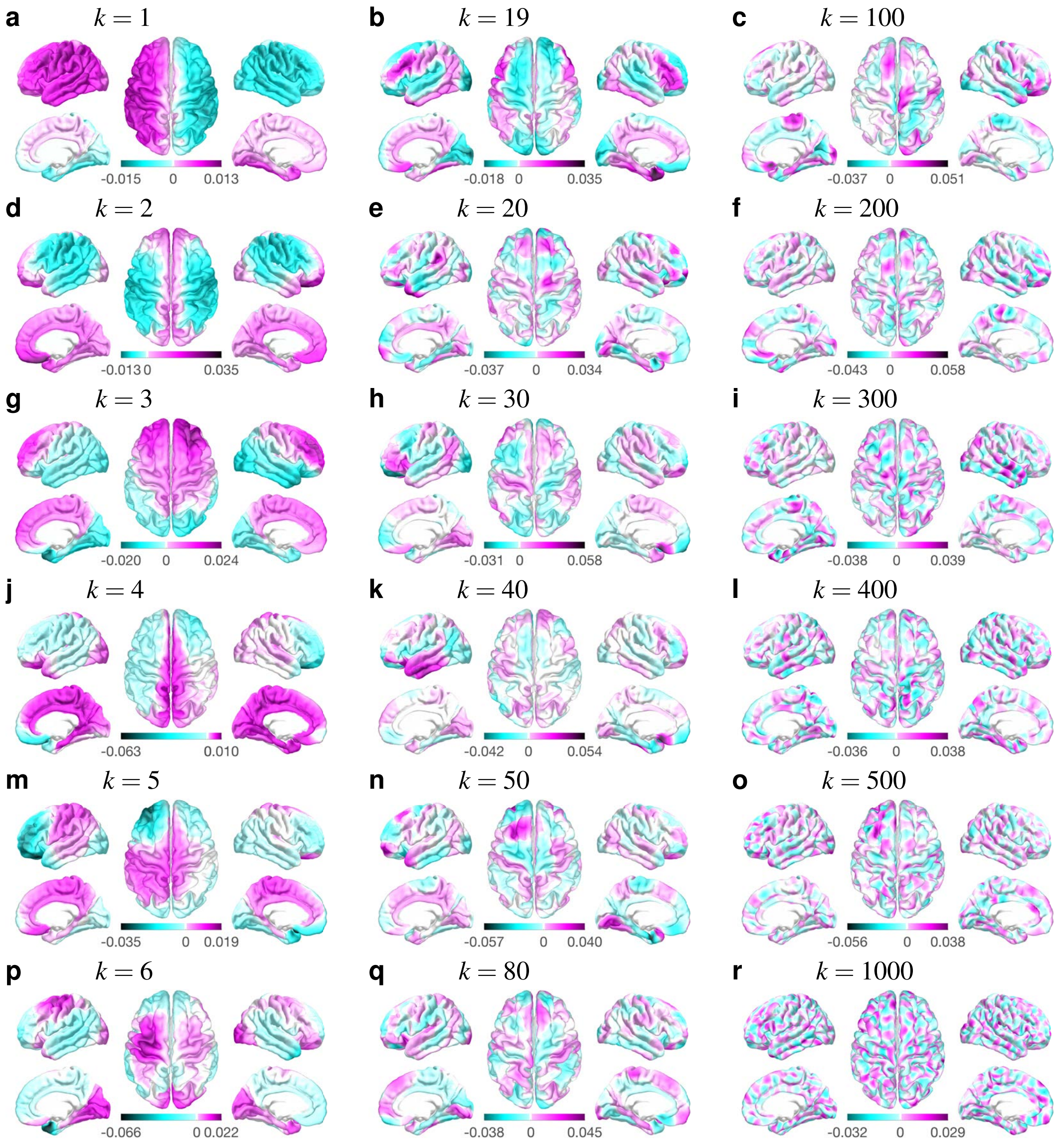

Figure S1. Examples of connectome harmonic patterns with increasing frequency (indicated by the spatial wavenumber k).

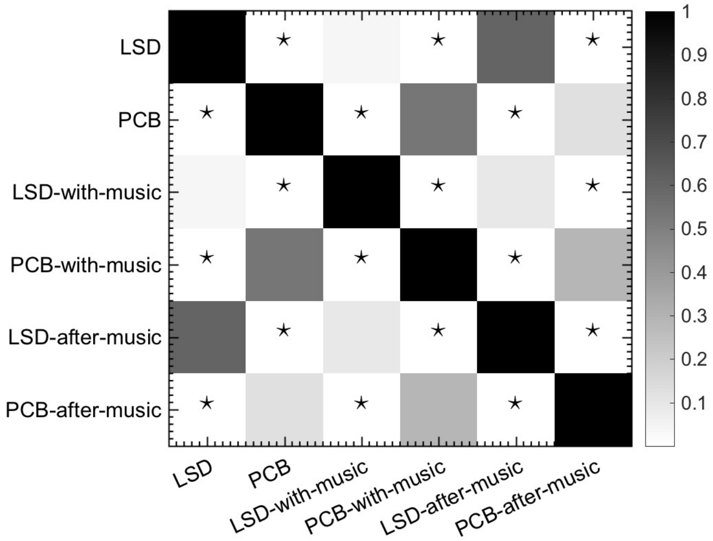

Figure S2. P-values of the two-sample Kolmogorov-Smirnov test performed between all pairs of conditions; i.e. all pairs between LSD, place (PCB), LSD-with-music, PCB-with-music, LSD-after-music, PCB-after-music, in order to test whether the probability distributions of energy values significantly differ in different conditions. All pairs of LSD vs. PCB conditions show significantly different distributions of energy values (with $\star : p < 10^{-85}$), whereas no pairs of LSD and no pairs of placebo conditions showed significant differences even in the case where one condition involved listening to music.).

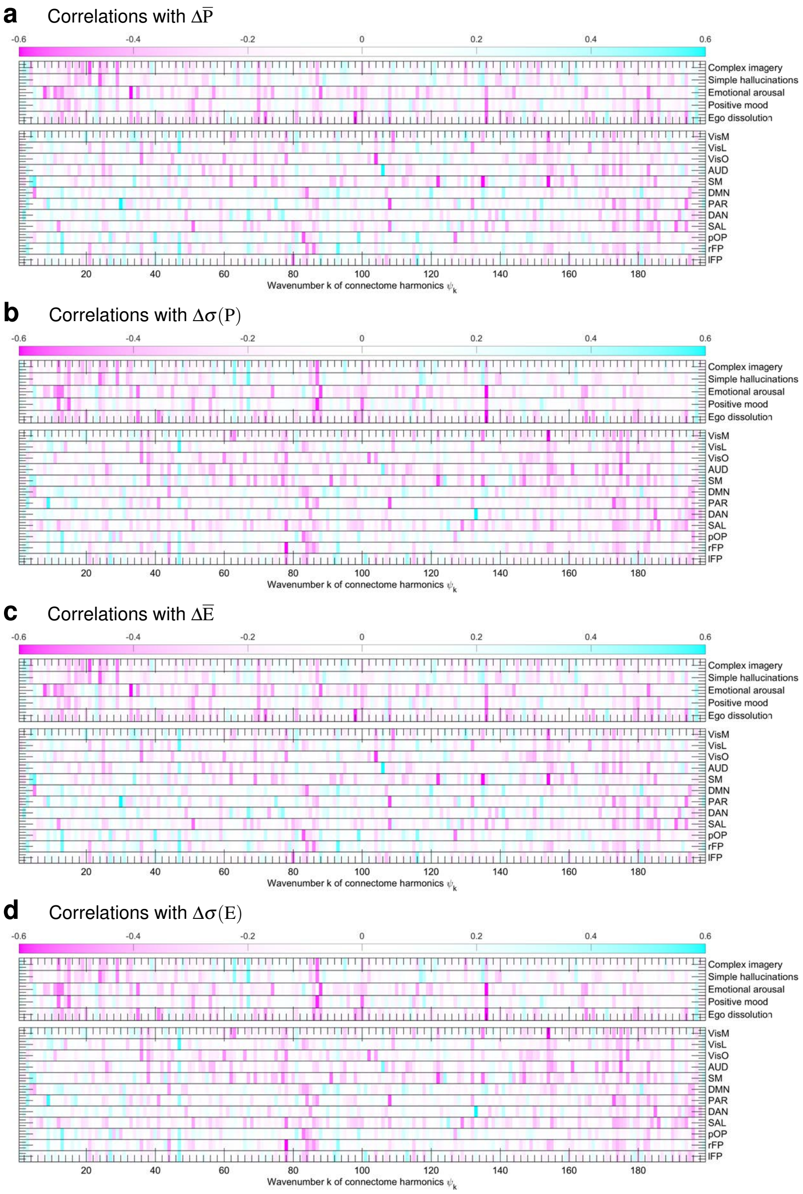

Figure S3. Partial correlations between the differences in (**a**) mean power, (**b**) power fluctuations, (**c**) mean energy and (**d**) energy fluctuations, for LSD and placebo conditions without music and subjective ratings (of simple hallucinations, complex imagery, emotional arousal, ego dissolution and positive mood) as well as the functional connectivity changes of different resting state networks.

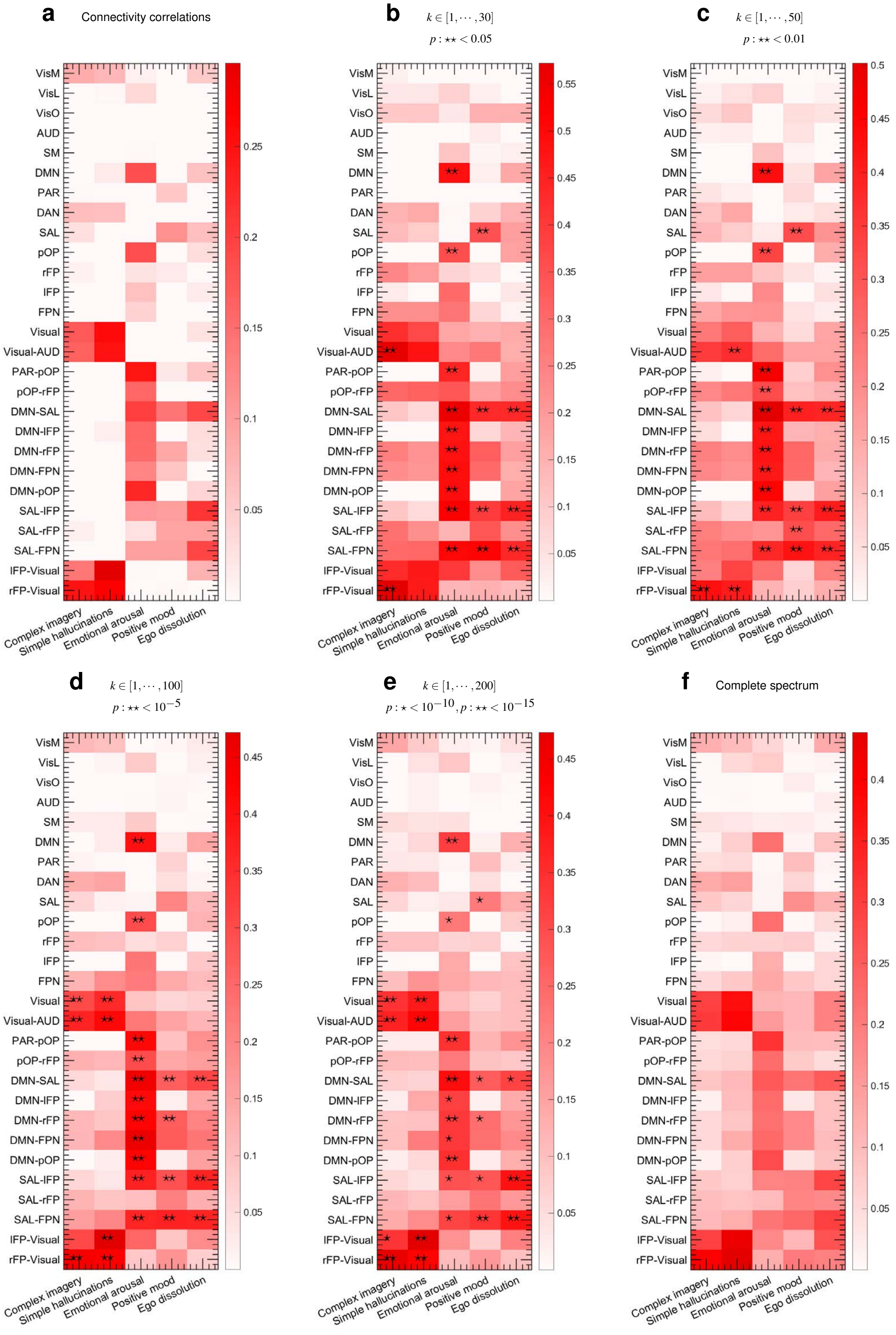

Figure S4. Multiple correlations between resting state network (RSN) functional connectivity changes and the ratings of subjective experiences. Multiple correlations are computed using (**a**) functional connectivity changes of the RSNs directly; expression of RSN connectivity changes with the first (**b**) 30, (**c**) 50, (**d**) 100, (**e**) 200 connectome harmonics and (**f**) complete connectome harmonic spectrum, respectively. Stars show significant correlations after Bonferroni correction.
